# Hydroxyapatite-Coated SPIONs and Their Influence on Cytokine Release

**DOI:** 10.3390/ijms22084143

**Published:** 2021-04-16

**Authors:** Bernhard Friedrich, Jean-Philippe Auger, Silvio Dutz, Iwona Cicha, Eveline Schreiber, Julia Band, Aldo R. Boccacccini, Gerhard Krönke, Christoph Alexiou, Rainer Tietze

**Affiliations:** 1 Department of Otorhinolaryngology, Head and Neck Surgery, Section of Experimental Oncology and Nanomedicine (SEON), Else Kröner-Fresenius-Stiftung-Professorship, Universitätsklinikum Erlangen, 91054 Erlangen, Germany; bernhard.friedrich@uk-erlangen.de (B.F.); iwona.cicha@uk-erlangen.de (I.C.); eveline.schreiber@uk-erlangen.de (E.S.); julia.band@uk-erlangen.de (J.B.); c.alexiou@web.de (C.A.); 2 Department of Internal Medicine 3—Rheumatology and Immunology, Universitätsklinikum Erlangen and Friedrich-Alexander University Erlangen-Nürnberg (FAU), 91054 Erlangen, Germany; Philippe.Auger@extern.uk-erlangen.de (J.-P.A.); Gerhard.Kroenke@uk-erlangen.de (G.K.); 3 Institute of Biomedical Engineering and Informatics, Technische Universität Ilmenau, 98693 Ilmenau, Germany; Silvio.Dutz@tu-ilmenau.de; 4 Institute of Biomaterials, Department of Materials Science and Engineering, FAU, 91058 Erlangen, Germany; aldo.boccaccini@ww.uni-erlangen.de

**Keywords:** hydroxyapatite, iron oxide nanoparticles, dexamethasone, lipopolysaccharides, cytokine release, macrophages

## Abstract

Hydroxyapatite- or calcium phosphate-coated iron oxide nanoparticles have a high potential for use in many biomedical applications. In this study, a co-precipitation method for the synthesis of hydroxyapatite-coated nanoparticles (SPION^HAp^), was used. The produced nanoparticles have been characterized by dynamic light scattering, X-ray diffraction, vibrating sample magnetometry, Fourier transform infrared spectrometry, atomic emission spectroscopy, scanning electron microscopy, transmission electron microscopy, selected area diffraction, and energy-dispersive X-ray spectroscopy. The results showed a successful synthesis of 190 nm sized particles and their stable coating, resulting in SPION^HAp^. Potential cytotoxic effects of SPION^HAp^ on EL4, THP-1, and Jurkat cells were tested, showing only a minor effect on cell viability at the highest tested concentration (400 µg Fe/mL). The results further showed that hydroxyapatite-coated SPIONs can induce minor TNF-α and IL-6 release by murine macrophages at a concentration of 100 µg Fe/mL. To investigate if and how such particles interact with other substances that modulate the immune response, SPION^HAp^-treated macrophages were incubated with LPS (lipopolysaccharides) and dexamethasone. We found that cytokine release in response to these potent pro- and anti-inflammatory agents was modulated in the presence of SPION^HAp^. Knowledge of this behavior is important for the management of inflammatory processes following in vivo applications of this type of SPIONs.

## 1. Introduction

Hydroxyapatite or calcium phosphate materials and coatings are used for a variety of different purposes including the removal of metal ions from contaminated water, as a redox luminescence switch, as well as in biomedical applications, e.g., the transfer of DNA into cells [1,2,3,4,5]. Moreover, hydroxyapatite is a suitable material for the coating of superparamagnetic iron oxide nanoparticles (SPIONs) to be used for hyperthermia in the treatment of tumors [6]. Such SPIONs have been proven to be biocompatible with only very low levels of cytotoxicity [6,7,8]. Due to their magnetic properties, SPIONs can also be directed to and enriched in certain areas in the body for targeted drug delivery purposes [9]. They can also be separated from media by simply using a magnet [10].

The current synthesis of SPIONs is carried out by different methods, mainly by co-precipitation or solvothermal synthesis [11,12,13]. Co-precipitation has the advantage of being quickly and easily carried out with a simple to control and adjustable setup. It has to be considered, however, that a one-step in situ co-precipitation of SPION^HAp^ bears the risk of producing iron-phosphates instead of coating the particles with a calcium phosphate or hydroxyapatite layer [14]. Consequently, it is useful to divide the synthesis into two steps, firstly the precipitation of iron oxide nanoparticles and secondly the precipitation of hydroxyapatite onto the surface of the iron oxide nanoparticles. Therefore, the nanoparticles must be stable in water-based media to be suitable for such a coating procedure. Citrate-coated SPIONs are an ideal precursor nanoparticle as they are very stable in water-based media and have been previously used in similar coating procedures [15,16,17].

Besides the direct use of hydroxyapatite-coated SPIONs, they can also be further modified with biomolecules including proteins, such as albumin, that can be linked to hydroxyapatite using bifunctional bisphosphonates, which increases the application field of such particles [18,19]. By binding proteins to particles, it is possible to link drugs onto this particle system [20]. The controlled release of drugs from biodegradable materials is an ongoing field of biomedical research [21].

The possibilities of binding the targeting or therapeutic agents and the interactions of particles with other components that occur in blood during disease or therapy, such as toxins and certain drugs, are still under investigation. Moreover, the cellular effects of SPION^HAp^ in combination with inflammatory and/or anti-inflammatory agents are not widely known or tested. Only a few studies mention selected iron oxide nanoparticles or calcium phosphate-coated SPIONs which can modulate the response of immune cells and may pose a potential threat as they induce cytokine release and inflammatory reactions [22,23]. This can be due to the fact that the particles can easily be contaminated with lipopolysaccharide (LPS) [22,24]. In particular, the functionalization steps pose a high risk of contamination, as LPS can be present in the used linker formulations or molecules, such as serum albumin used in this process [25]. The interaction of LPS and nanoparticles has been barely tested but has high importance for their safe application. LPS can be present in the blood, as a result of contaminated food uptake, or in the special case of sepsis and other infections with Gram-negative bacteria [26,27]. Furthermore, the interactions of nanoparticles with toxins/drugs should be considered if they are expected to be used in an in vivo environment, where they can encounter immune cells. Circulating nanoparticles larger than 50 nm in diameter are rapidly scavenged by macrophages and hence enriched in macrophage-rich tissues. Macrophages, rather than other immune cells, are known for the avid uptake of particles, which are expected to affect their biology. It is particularly important to investigate how the presence of LPS in combination with used nanoparticles can modulate their response in terms of cytokine release. Furthermore, it is an important question, whether this response can be suppressed by anti-inflammatory medication [28]. In this context, dexamethasone is a well-known drug prescribed to suppress inflammatory reactions which has recently gained wider attention thanks to its potential in COVID-19 therapy [29,30,31].

The aim of this study was to investigate the effects of hydroxyapatite-coated SPIONs on cytokine release from macrophages under cell culture conditions. The SPION^HAp^ synthesized using an adapted co-precipitation method [6] have been tested for their cytotoxicity by a well-established flow-cytometric method. The release of the pro-inflammatory cytokines, tumor necrosis factor alpha (TNF-α), and interleukin-6 (IL-6), by murine macrophages, was investigated in vitro in response to SPION^HAp^. It was subsequently investigated to what extent this can be further modulated by the presence of the inflammatory and anti-inflammatory agents LPS and dexamethasone, respectively.

## 2. Results and Discussion

### 2.1. Characterizations of the Materials

**Figure 1:**
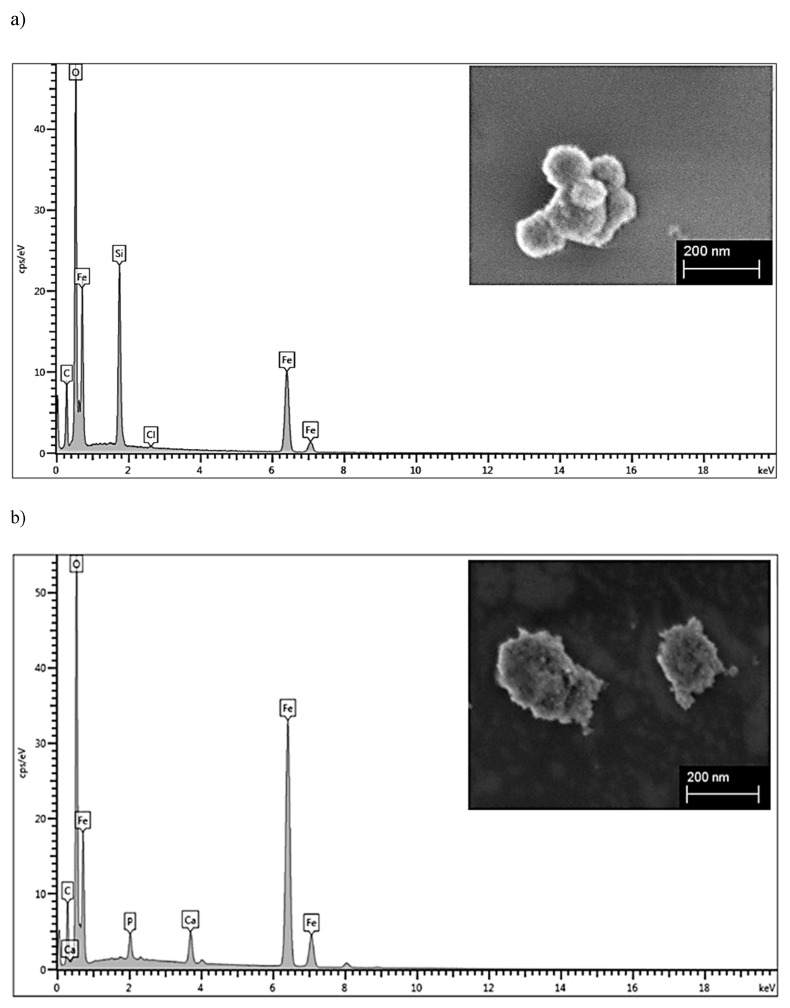
SEM images of (**a**) SPION^Cit^ and (**b**) SPION^HAp^ and corresponding EDX signal.

For SPION^HAp^, a two-step procedure with intermediate citrate coating of iron oxide nanoparticles (SPION^Cit^) was used as described in [6,17]. The use of citrate-coated nanoparticles had advantageous effects on the synthesis. Most importantly, the particles were stable and did not agglomerate while the pH was increased during the coating with hydroxyapatite in the second step. Figure 1a shows that the freeze-dried SPION^Cit^ had a diameter of about 90 to 120 nm and the SPION^HAp^ in b) had a diameter of 190 nm according to scanning electron microscopy (SEM). This was similar to the z-average of the hydrodynamic diameter, which was found to be around 190 nm (Table 1 and Figure A2) at neutral pH, with a relatively narrow size distribution according to the polydispersity index. In addition, a sponge-like structure on the sphere-shaped nanoparticles was observed in the SEM images, as also reported in previous studies [8]. The hydrodynamic size determined by dynamic light scattering (DLS) evaluation shows large variability in the case of hydroxyapatite-coated iron oxide nanoparticles obtained by other techniques. For example, the most common solvothermal method produces particles with a diameter of around 95 nm as described in [6] and up to 570 nm [2]. As an indicator of particle stability, the zeta potential (Table 1) was measured. An increase in zeta potential from −46.9 mV (SPION^Cit^) to −28.1 mV for SPION^HAp^ was detected after the coating procedure. Both particle types showed good stability in water-based media showing minimal precipitation after 48 h.

**Figure 2:**
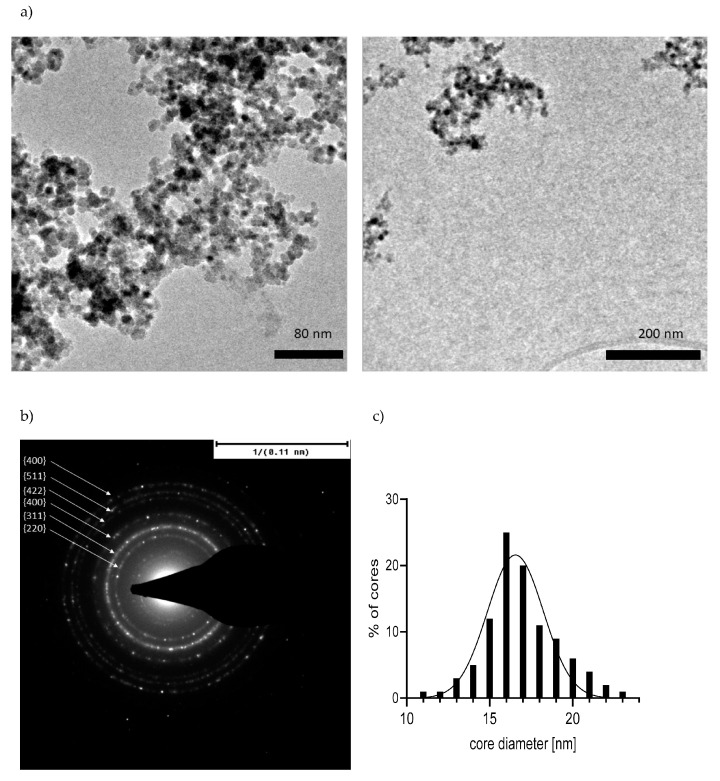
(**a**) TEM images of SPION^HAp^; (**b**) selected area diffraction; (**c**) core size distribution.

TEM images (Figure 2) of SPION^HAp^ showed a typical structure for SPIONs synthesized by co-precipitation methods, showing a multicore structure consisting of several smaller cores [16]. The size of these individual cores was found to be 16.5 nm on average (Figure 2A,C) comparable to the size in [2], where the size of the initial iron oxide particles before the coating was found to be between 100 to 500 nm, and the aggregated cores were 10 to 20 nm in size.

The energy-dispersive X-ray spectroscopy (EDX) signal (Figure 1b) from the SPION^HAp^ evidenced the presence of iron, oxygen, carbon, phosphorus, and calcium with a ratio of Ca/P of 1.65, which is in accordance with hydroxyapatite. In comparison, only iron, oxygen, and carbon but no Ca or P were found in the EDX signal of SPION^Cit^ (a silicon peak is present because the sample was prepared on a silica wafer). Additionally, a layer of the SPION^HAp^ was prepared by evaporation of a nanoparticle dispersion and an EDX-Mapping (Figure A3) was performed, showing a homogeneous distribution of the expected elements.

The selected area diffraction of SPION^HAp^ showed typical rings, indicating polycrystalline structure composed of small grains (Figure 2B). Six rings are visible, corresponding to lattice planes as follows, starting from the center of the diffraction pattern: {220}, {311}, {400}, {422}, {511}, and {440}, matching the diffraction pattern of iron oxide [32,33,34].

**Figure 3:**
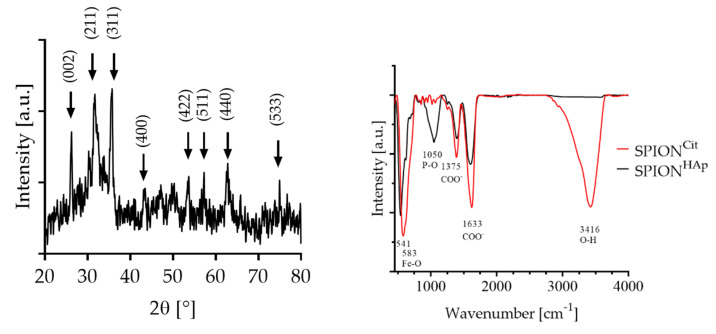
Right: XRD of SPION^HAp^ with magnetite/maghemite-related characteristic peaks at 2*θ* = 35.6°, and for hydroxyapatite/hydroxyapatite at 2*θ* = 32.4°. Left: FTIR-spectra of SPION^Cit^ with a Fe-O peak at 541 cm^−1^ and SPION^HAp^ with a Fe-O peak at 583 cm^−1^ and P-O at 1050 cm^−1^.

The X-ray diffraction (XRD) pattern shown in Figure 3 confirmed the presence of magnetite/maghemite-related characteristic peaks according to (311) and (422) as well as (440) refractions. The most dominant peak for calcium phosphate/hydroxyapatite-coated nanoparticles was obtained at 2*θ* = 32.4°, corresponding to the (211) crystal plane, as well as the peak at 2*θ* = 26.8°, 46° and 49° [35]. This diffraction pattern corresponds to previously reported patterns obtained by solvothermal synthesis followed by a precipitation of hydroxyapatite [2,6,36]. The SPION^Cit^ that was used did not show the presents of peaks that could be related to phosphate/hydroxyapatite [16]. From the values obtained by the XRD, the lattice parameter and crystallite size of SPION^HAp^ were calculated to be 8.4 Å for magnetite using the peak at 35.4° [32]. The crystallite size was found to be about 16 nm for magnetite corresponding to the values obtained by the TEM images. Furthermore, the selected area diffraction of the SPIONs confirmed the presence of magnetite/maghemite according to the ring pattern as shown in Figure 2B [32,33,34].

The Fourier transform infrared (FTIR)-spectrum of SPION^HAp^ showed a strong band at 1050 cm^−1^, corresponding to the presence of PO_4_^−3^ [37]. The dominant band for all particles is the Fe-O stretching band at 541 to 581 cm^−1^. SPION^Cit^ and SPION^HAp^ showed deprotonated carboxylic acid groups (COO^-^) at broad bands around 1375 and 1633 cm^−1^ [38,39]. These peaks were less pronounced for SPION^HAp^. This can be explained by the coating process, as the procedure reactions and washing steps lead to a reduction in the citrate amount on the nanoparticles.

**Figure 4:**
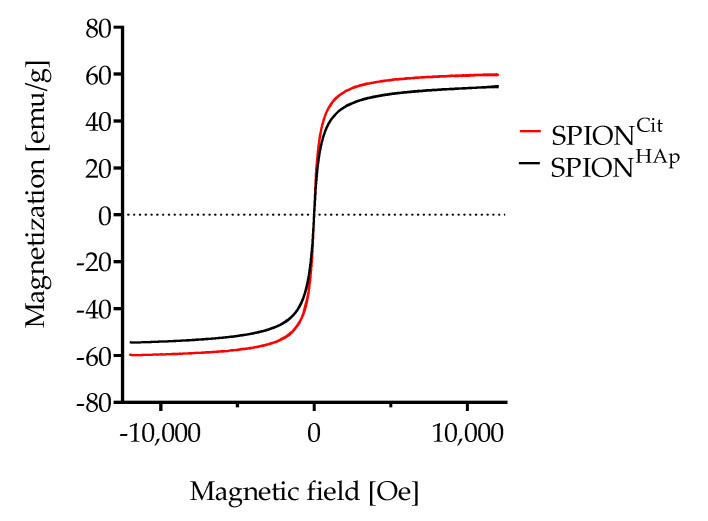
Magnetization curves of SPION^Cit^ and SPION^HAp^.

**Figure A1:**
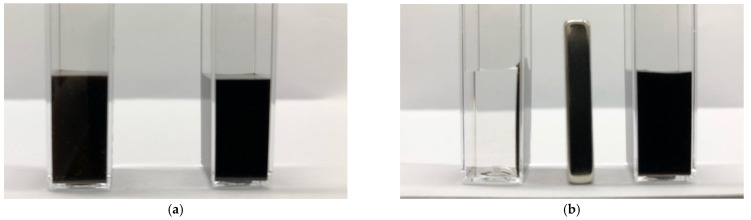
(**a**) Separation of SPION^Cit^ (left) compared to SPION^HAp^ (right) before separation. (**b**) Separation of SPION^Cit^ (left) compared to SPION^HAp^ (right) after 30 sec of magnetic separation.

**Figure A2:**
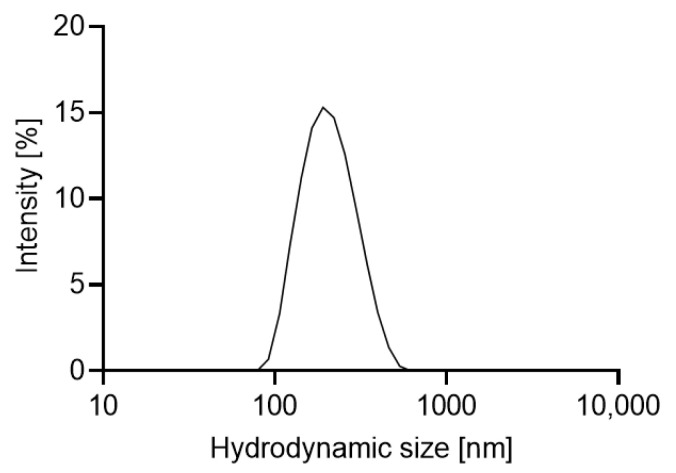
Size distribution of SPION^HAp^ obtained by dynamic light scattering.

**Figure A3:**
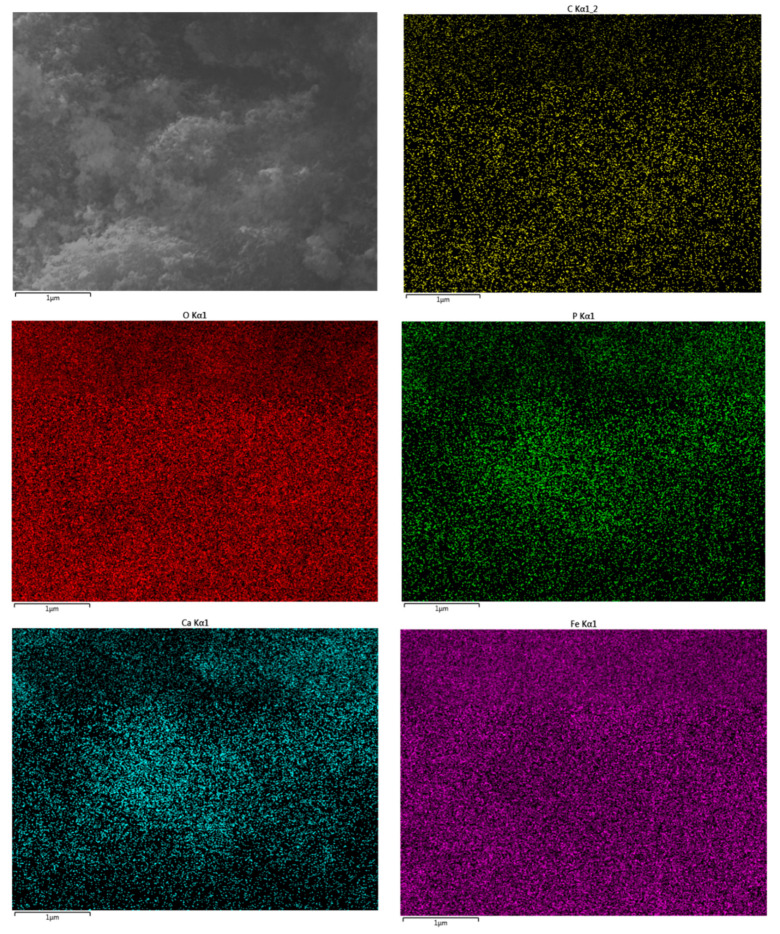
EDX-Mapping of the SPION^HAp^ after drying dispersed nanoparticles on air. The Kα of carbon (yellow), oxygen (red), phosphor (green), calcium (blue), and iron (violate) and are shown.

As shown in Figure 4, SPION^Cit^ and SPION^HAp^ exhibited superparamagnetic behavior. A minor difference in their saturation magnetization could be observed. After the coating step, the saturation magnetization decreased from 59.7 emu/g for citrate-coated particles to 54.7 for SPION^HAp^, which equals a reduction of 9%. A similar decrease has also been seen in the volumetric susceptibility of the samples. The volume susceptibility of SPION^HAp^ at a concentration of 1 mg Fe/mL was found to be 5.46 × 10^−3^ ± 0.09 × 10^−3^ χ_v_ in comparison to SPION^Cit^ with a higher magnetic susceptibility of 6.30 × 10^−3^ ± 0.04 × 10^−3^ χ_v_, which corresponds to a reduction of 14%. In comparison to SPION^Cit^, it was possible to separate SPION^HAp^ with a high efficiency from water-based media within a few seconds (Figure A1).

### 2.2. Cytotoxicity Testing

Being very resistant to toxic stimuli, macrophages are not the best candidate cell type for toxicity testing. Additionally, they adhere to surfaces, and trypsinization, which is required for the FACS analysis, reduces the cell viability drastically, which may influence the read-outs [40]. For this reason, we used several types of suspension cell lines, including EL4 mouse cells, THP-1 monocytic cells, and Jurkat cells (Figure A5) for toxicity evaluation [41]. The influence of SPION^HAp^ on cell viability was tested for concentrations ranging from 25 to 400 μg Fe/mL, as previously described [42,43]. AxV and propidium iodide (PI) were used to differentiate between viable (AxV-PI-, green), early apoptotic (AxV+PI-, blue), and late apoptotic/necrotic (PI+, red) cells.

**Figure 5:**
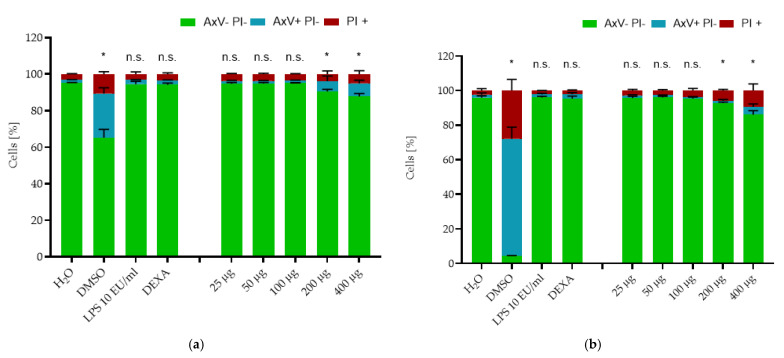
Effects of SPION^HAp^ on the viability of EL4 cells (ATCC^®^ TIB-39™). Cells were incubated with SPION^HAp^ particle concentrations of 25 to 400 μg/mL and additional controls of H_2_O, DMSO 2%, dexamethasone (DEXA) 0.1 µM, and LPS. After (**a**) 24 and (**b**) 48 h, cells were stained with Annexin A5 FITC conjugate (AxV) and propidium iodide (PI) to detect apoptotic and necrotic cells by flow cytometry. Viable cells (AxV- PI-) are visualized in green, apoptotic cells (AxV+ PI-) in blue and necrotic cells (PI+) in red. Each experiment was performed in triplicate. The mean values are shown with SD. Significance between treatment groups and control (viable cells) at the respective time is indicated by asterisks: * *p* < 0.05. *p* values higher than 0.05 were assumed to be not significant (n.s.).

**Figure A4:**
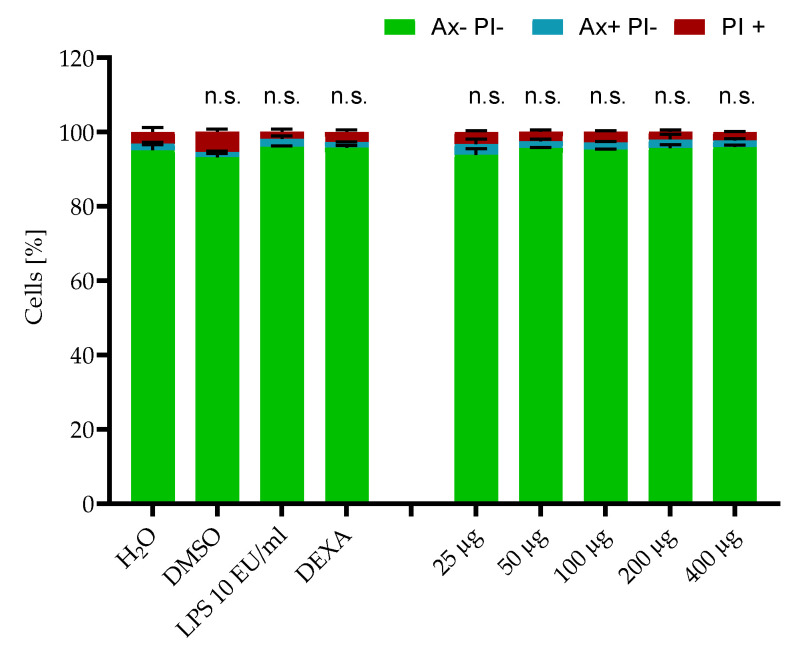
Effects of SPION^HAp^ on the viability of EL4 cells (ATCC^®^ TIB-39™). Cells were incubated with SPION^HAp^ particle concentrations of 25 to 400 μg/mL and additional controls of H_2_O, DMSO 5%, dexamethasone (DEXA) 0.1 µM, and LPS. After 0 h cells were stained with Annexin A5 FITC conjugate (AxV) and propidium iodide (PI) to detect apoptotic and necrotic cells by flow cytometry. Viable cells (AxV- PI-) are visualized in green, apoptotic cells (AxV+ PI-) in blue, and necrotic cells (PI+) in red. Each experiment was performed in triplicate. The mean values are shown with SD. Significance between treatment groups and control (viable cells) at the respective time is indicated by asterisks: * *p* < 0.05. *p* values higher than 0.05 were assumed to be not significant (n.s.).

The toxicity directly after administration of the nanoparticles (0 h) is shown in Figure A4. Only a minor decrease in viability was detected in the DMSO control, where the tested viability was 93.09% ± 1.0%, compared to the H_2_O control with 95.01% ± 1.5%. None of the other tested samples showed any decrease in cell viability at this time point. Figure 5 shows the effects of SPION^HAp^ on EL4 cells after 24 h and 48 h of incubation. After 24 h, the percentage of the viable cells in negative control samples (water) was still at 95.0% ± 0.04%. This amount decreased for the DMSO-treated cells to 65.23% ± 4.47%, whereby 24.09% ± 3.19% were apoptotic cells with a damaged plasma membrane and about 10.6% ± 1.3% were necrotic cells. The viability at 25, 50 and 100 μg Fe/mL did not significantly decrease after 24 h of incubation. However, the number of viable cells was significantly reduced to 90.5 ± 1.0% at 200 μg Fe/mL and to 87.7 ± 1.6% at 400 μg Fe/mL. In addition for the EL4 the membrane potential was analysed at this timepoint. Figure A6 shows that the values obtained in Figure 5 are comparable. The amount of Dil+ and Dil- negative cells is matching to that of the AxV PI staining.

**Figure 6:**
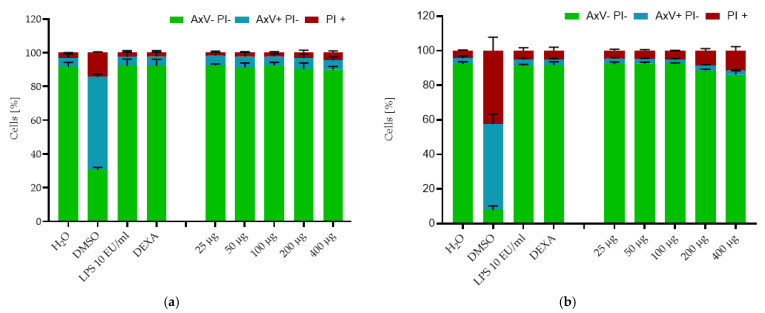
Effects of SPION^HAp^ on the viability of THP-1 (ATCC^®^ TIB-202™). Cells were incubated with SPION^HAp^ particle concentrations of 25 to 400 μg/mL and additional controls of H_2_O, DMSO 2%, dexamethasone (DEXA) 0.1 µM, and LPS. After (**a**) 24 and (**b**) 48 h of incubation. Each experiment was performed in triplicate. The mean values are shown with SD. Significance between treatment groups and control (AxV- PI-) at the respective time is indicated by asterisks: * *p* < 0.05. *p* values higher than 0.05 were assumed not significant (n.s.).

At the last time point (48 h), only 4.3 ± 0.2% viable cells were detected in the DMSO control, as the number of apoptotic cells increased to 67.7% and of necrotic cells to 27.9% compared to the water control, where cell viability was 95.87% ± 1.1%. The viability of cells treated with the tested SPION^HAp^ showed no decrease at the concentrations of 25, 50, or 100 μg Fe/mL. At the concentration of 200, we detected a minimal decrease to 92.7% ± 0.6%, and a stronger reduction of viability to 86.0% ± 2.3% at 400 µg/mL Fe. Thus, SPION^HAp^ at this range of concentrations showed only a minimal dose-dependent toxicity at 24 and 48 h. Similar values were obtained by testing THP-1 cells with the same nanoparticles and method (Figure 6) after 24 and 48 h of incubation.

The side scatter of El4 and THP-1 cells after 24 h of incubation are shown in Figure A7. El4 did not show a dose-dependent increase in side scatter as they are non-phagocytotic cells. In comparison, the THP-1 showed an increase in side scatter, which can be explained by to the uptake of SPION^HAp^.

**Figure A5:**
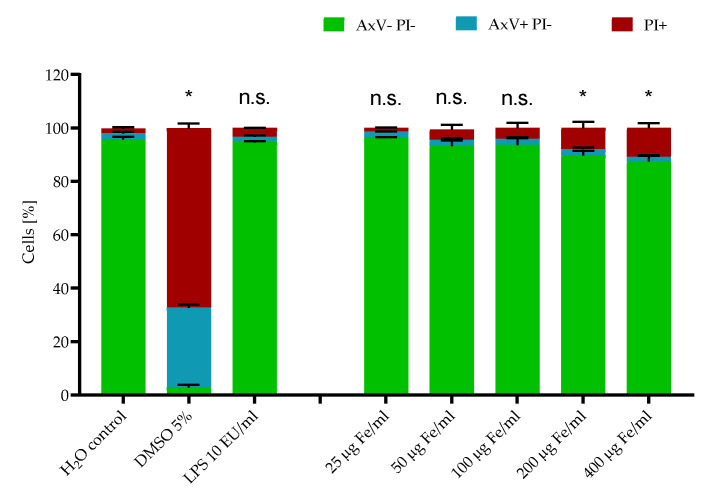
Effects of SPION^HAp^ on the viability of Jurkat cells (ACC 282). Cells were incubated with SPION^HAp^ particle concentrations of 25 to 400 μg/mL. After 48 h, cells were stained with Annexin A5 FITC conjugate (AxV) and propidium iodide (PI) to detect apoptotic and necrotic cells by flow cytometry. Viable cells (AxV- PI-) are visualized in green, apoptotic cells (AxV+ PI-) in blue, and necrotic cells (PI+) in red. The experiments were performed in triplicate. The mean values are shown with SD. Significance between treatment groups and control (viable cells) at the respective time is indicated by asterisks: * *p* < 0.05. *p* values higher than 0.05 were assumed to be not significant (n.s.).

**Figure A6:**
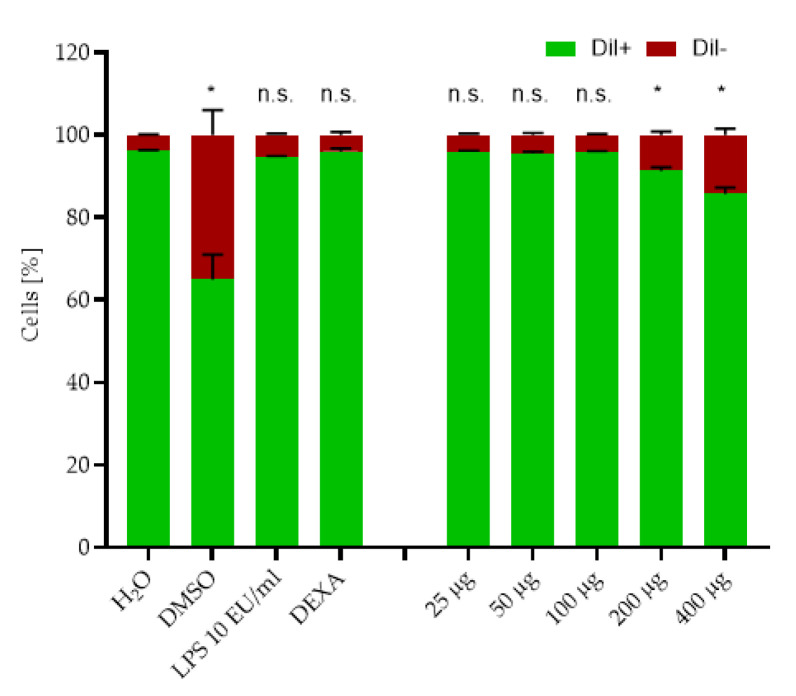
Effects of SPION^HAp^ on viability of EL4 cells (ATCC^®^ TIB-39™). Cells were incubated with SPION^HAp^ particle concentrations of 25 to 400 μg/ml and additional controls of H_2_O, DMSO 5%, dexamethasone (DEXA) 0.1 µM and LPS. After 24 h cells were stained with Dil-Hoe and evaluated by flow cytometry. Viable cells (Dil+) are visualized in green, apoptotic cells and necrotic cells (Dil-) in red. Each experiment was performed in triplicates. Shown are the mean values with SD. Significance between treatment groups and control (viable cells) at the respective time is indicated by asterisks: * *p* < 0.05. *p* values higher than 0.05 were assumednot significant (n.s.).

**Figure A7:**
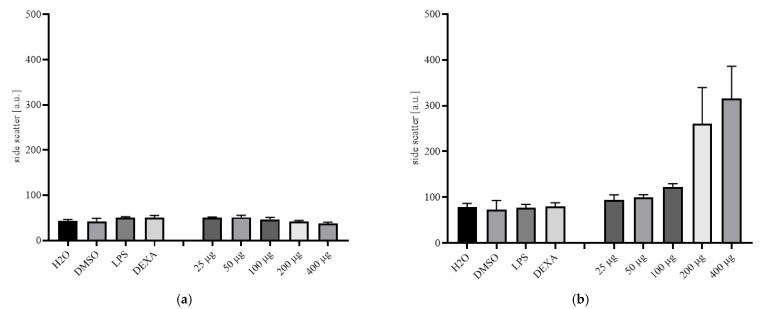
Effects of SPION^HAp^ on side scatter after 24 h of incubation on (**a**) El4 cells (ATCC^®^ TIB-39™) and (**b**) THP-1 THP-1 (ATCC^®^ TIB-202™).

Furthermore, similar results were also obtained in Jurkat cells after 48 h of incubation, as shown in Figure A5. After 48 h, the percentage of viable cells in the negative control samples was still at 95.6% ± 1.0%. This amount decreased in the positive control samples (DMSO 5%) to 2.9%. In the DMSO control, the number of apoptotic cells increased to 29.9% and of necrotic cells to 67.2%, compared to the water control after 48 h. The viability of cells treated with the tested SPION^HAp^ showed no decrease at a concentration of 25 μg Fe/mL, and only a minor reduction in viability to 93.1% was found at 100 μg Fe/mL. At a concentration of 400 µg/mL Fe, we detected a slight further decrease to 87.5% ± 2.3% compared to the untreated control. Thus, for the tested particles at a range of concentrations, only a minimal dose-dependent toxicity was observed after 48 h in three different cell types. The toxicity of SPION^Cit^ was evaluated in previous studies showing higher toxicity compared to the as-prepared SPION^HAp^ [15,44].

### 2.3. Cytokine Release Study

**Figure 7:**
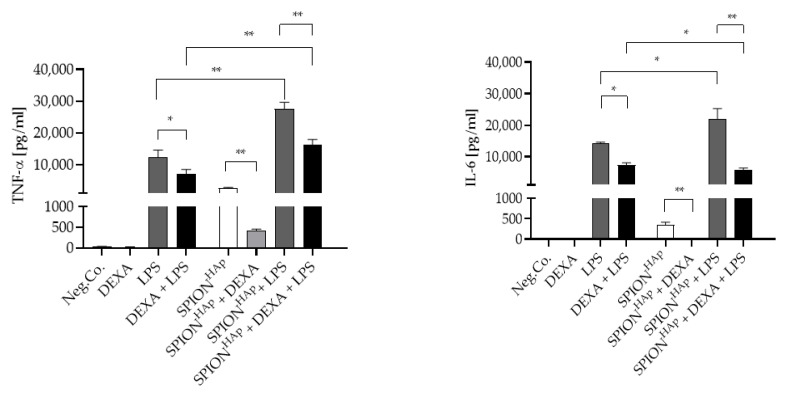
TNF-α and IL-6 release from murine macrophages after treatment with SPION^HAp^ at an iron concentration of 100 µg/mL without or with dexamethasone (DEXA) and/or LPS. Neg.Co corresponds to untreated cells. Experiments were performed in triplicate. Significance between treatment groups and control at the respective time is indicated by asterisks: * *p* < 0.05, ** *p* < 0.005. *p* values higher than 0.05 were assumed not significant (n.s.).

To ensure that no initial endotoxin contamination was influencing the results, a factor C test was carried out on the nanoparticles prior to the cytokine release study. The test did not detect any endotoxin in the synthesized SPION^HAp^ batches. We, therefore, excluded the influence of endotoxin contamination on the cytokine release study. The release of the pro-inflammatory cytokines TNF-α and IL-6 from macrophages under different conditions is shown in Figure 7. For this study, an amount of SPION^HAp^ corresponding to an Fe concentration of 100 µg/mL was used. SPION^HAp^ alone induced a slight release of TNF-α and minimal release of IL-6 from the cells. This release possibly contributes to the minor cytotoxic effects induced by hydroxyapatite-coated nanoparticles, as shown in previous studies [6,8]. Furthermore, calcium phosphate particles have been shown to modulate cytokine release, likely due to the fact that calcium is needed for cytokine production [23]. The release of TNF-α caused by the SPION^HAp^ alone was significantly reduced by the addition of dexamethasone (0.1 µM). For IL-6, a complete abolition of the SPION^HA^-induced release was observed in presence of dexamethasone, leading to the assumption that SPION^HA^-induced cytokine release can be suppressed by anti-inflammatory agents.

Incubation of murine macrophages with 10 EU/mL of LPS led to a strong release of TNF-α and an even larger release of IL-6. This is in accordance with previous studies showing that cytokine release is strongly dependent on the number of LPS, with a higher impact on IL-6 than on TNF-α [45,46]. As expected, dexamethasone significantly reduced the LPS-induced release of both cytokines.

Co-stimulation of nanoparticle-treated samples with 10 EU/mL LPS had a synergistic effect on cytokine release in comparison with either treatment alone, with an over 4.5-fold increase in TNF-α release, and about a 55% increase in IL-6. This effect can be explained by the simultaneous extracellular and intracellular co-stimulation of cells by LPS [47], via the Toll-like receptor 4, and by the nanoparticles, following phagocytosis.

In comparison with macrophages treated with both LPS and SPION^HAp^, co-treatment with dexamethasone resulted in a strong decrease in cytokine release. This effect was different as observed in the absence of SPIONs, and the reduction was relatively similar for IL-6 (47.5% reduction) and TNF-α (42.1% reduction). These results might be caused by an increased uptake of dexamethasone due to the presence and the subsequent phagocytosis of SPION^HAp^. Finally, the anti-inflammatory effects of dexamethasone on LPS-induced cytokine release were potentiated in the presence of SPION^HAp^, with a greater effect on IL-6 (74.0% reduction) than on TNF-α (59.7% reduction). Further, the toxicity tests showed that the particles themselves had almost no effects on the tested cell lines, it was assumed that the main cause of cytokine changes is caused by the addition of dexamethasone and LPS. Given these results, testing of nanoparticles for effects on cytokine release is recommended before considering their application in vivo, in order to detect possible side effects and influences. Besides the unwanted side effects, our results could be relevant for the application of SPION^HAp^ in certain disease conditions. For example, in dexamethasone-treated patients that suffer from inflammatory or autoimmune diseases, an additional reduction of IL-6 release could be beneficial. Furthermore, the stimulation of cytokine production could be an advantage for cancer treatment, as it stimulates the immune response and could lead to an increased recruitment of immune cells. As SPION^HAp^ can accumulate in certain macrophage-rich areas, this effect could support the treatment.

## 3. Materials and Methods

### 3.1. Materials

Iron (II) sulfate heptahydrate and iron (III) chloride hexahydrate were purchased from Merck KGaA (Darmstadt, Germany). Propidium iodide (PI), disodium ethylenediaminetetraacetic acid, LPS from Escherichia coli O11:B4, and dexamethasone were obtained from Sigma-Aldrich Chemie GmbH (Darmstadt, Germany). Sodium citrate, ammonia 25% (*v*/*v*), ammonium chloride, potassium bicarbonate, calcium chloride dihydrate, sodium hydroxide, calcium nitrate tetrahydrate, di-ammonium hydrogen phosphate potassium bromide (KBr) (spectroscopy grade), and dimethyl sulfoxide (DMSO, 99.5%) were purchased from Carl Roth GmbH & Co.-KG (Karlsruhe, Germany). Minisart NML syringe filters (0.2 μm pore size), Dulbecco’s Modified Eagle’s Medium (DMEM), Hoechst 33342 (Hoe), Annexin A5 fluorescein isothiocyanate (FITC) conjugate (AxV), and 1,1′-dimethyl-3,3,3′,3′-tetramethylindodicarbocyanineiodide (DiI) were obtained from Thermo Fisher Scientific (Waltham, MA, USA). RPMI 1640 medium supplemented with 10% fetal calf serum (FCS), 1% L-glutamine and penicillin streptomycin-solution 5000 U/mL, GlutaMAX supplement were obtained from Live Technologies (Carlsbad, USA), T cell leukemia cells Jurkat (ACC 282), THP-1 (ATCC^®^ TIB-202™), and EL4 (ATCC^®^ TIB-39™) were obtained from DSMZ (German Collection of Microorganisms and Cell Cultures, Braunschweig, Germany), and Ringer’s solution from Fresenius Kabi (Bad Homburg, Germany). Deionized water was obtained from a Siemens Ultra Clear system (Evoqua Water Technologies, Guenzburg, Germany).

### 3.2. Synthesis of Hydroxyapatite-Coated Iron Oxide Nanoparticles (SPION^HAp^)

The synthesis of hydroxyapatite-coated SPIONs was performed in a two-step procedure. We first used a modified protocol to synthesize citrate-coated iron oxide nanoparticles. Briefly, iron (II) sulfate and iron (III) chloride (ratio 1:2) were dissolved in 50 mL water and stirred at 250 rpm under an argon atmosphere to prevent oxidation. Iron oxide was precipitated by the addition of ammonia solution. After 10 min of stirring, a solution bearing sodium citrate was added, and the mixture was stirred for 30 min at 90 °C and 450 rpm. To remove excess sodium citrate, the cooled SPION^Cit^ were sterile filtered through a 0.2 μm pore diameter syringe filter and dialyzed against water with 100 kDa Spectra/Por^®^ dialysis tubes from Repligen (Waltham, MA, USA). The dialyzed SPION^Cit^ were kept in water at 4 °C until further use.

SPION^HAp^ were subsequently synthesized using a method described before in [6]. Briefly, SPION^Cit^ containing 160 mg Fe were dispersed in water and 60 mg of calcium nitrate tetrahydrate were added to the dispersion. The pH of the dispersion at 40 °C was adjusted to 9.1 by the addition of NaOH (1 N). After 10 min, 30 mg of di-ammonium hydrogen phosphate were dissolved in 5 mL water and then added to the dispersion. The dispersion was kept at 40 °C for 1 h and 250 rpm before it was cooled to room temperature and magnetically washed three times with water. After separation, the particles were dried at 60 °C for 12 h and redispersed in endotoxin-free water afterward. The final dispersion was stored at 4 °C until further use.

### 3.3. Iron Quantification

For the determination of the iron concentration, atomic emission spectroscopy (AES) was used (Agilent 4200 MP-AES, Agilent Technologies, Santa Clara, CA, USA). A commercially available iron solution (1.000 mg/L, Bernd Kraft, Duisburg, Germany) served as an external standard. Samples were diluted at a ratio of 1:20. To 20 μL of the diluted sample, 80 μL of 65% nitric acid was added. The mixture was heated to 95 °C for 10 min and afterward diluted with water to 2 mL. Triplicate measurements were carried out at a wavelength of 371.993 nm and the results were averaged.

### 3.4. Hydrodynamic Particle Size and Zeta Potential

Dynamic light scattering (DLS) was performed with a Zetasizer Nano ZS (Malvern Panalytical, Almelo, Netherlands) to determine the hydrodynamic particle size of the SPIONs at 25 °C in water (refractive index 1.33, viscosity 0.8872 mPa·s, backscattering mode at 173°). The same device was used to measure the particle’s aqueous zeta potential with 78.5 as dielectric constant. Measurements were done in triplicate at an iron concentration of 50 µg/mL and pH of 7.4 and the results were averaged.

### 3.5. SEM Imaging

SEM images of the particles were taken with a Zeiss Auriga SEM (Carl Zeiss, Oberkochen, Germany) operated at an acceleration voltage of 3 kV. Samples were diluted to an iron concentration of 100 µg/mL, dropped on a silica wafer or alumina sample holder, and lyophilized prior to the microscopic analysis.

### 3.6. Energy-Dispersive X-ray Analysis (EDX)

The composition of SPION^Cit^ and SPION^HAp^ was studied using energy-dispersive X-ray analysis. The EDX detector (Silicon Drift Detector (SDD)—X-MaxN, Oxford Instruments) was integrated into the Zeiss Auriga SEM (Carl Zeiss, Oberkochen, Germany). Samples observed with the SEM were therefore used for the EDX analysis, whereby the acceleration voltage was increased to 18 keV.

### 3.7. Magnetic Susceptibility

As an indicator for the magnetizability of SPIONs, the magnetic susceptibility was measured with an MS2G magnetic susceptibility meter (Bartington Instruments, Oxfordshire, UK). The SPIONs were diluted to an iron concentration of 1 mg/mL in H_2_O prior to measurement.

### 3.8. X-ray Diffraction Analysis

A Rigaku MiniFlex 600 diffractometer was used to perform a *θ*/2*θ* XRD analysis to determine the phases and crystal structures present in the specimen. Powders were obtained from particle dispersions by lyophilization at 1 mbar (Alpha 1–2 LDplus, Martin Christ Gefriertrocknungsanlagen, Osterode am Harz, Germany). Powders were placed on a specimen holder and softly pressed. A Cu-Kα1 beam was used as the X-ray source (wavelength *λ* = 1.54059 nm) and the angular range was 20 to 80° with a step size of 0.03°/s. Indexation of the peaks was performed according to previous reports [35]. The distance of the atomic planes dhkl calculated by Bragg’s law Equation (1) together with Equation (2) was used to receive the lattice parameter of the samples.

Where *λ* is the wavelength, *θ* is the diffraction angle, and *h*, *k*, *l* are the Miller indices of the diffraction plane. With the usage of the Debye–Scherrer formula Equation (3), the crystallite size *d_cry_* of the samples was calculated.

(1) $$d_{hkl}=\frac{\lambda }{2*\sin\left(\theta \right)}$$

(2) $$d_{hkl}=\frac{a}{\sqrt{h^{2}+k^{2}+l^{2}}}$$

where FWHM represents the full-width at the half-maximum value of the XRD peaks.

(3) $$d_{cry}=\frac{0.9\;*\;\lambda }{FWHM\;*\;\cos\left(\theta \right)}$$

### 3.9. Vibrating Sample Magnetometry (VSM)

Freeze-dried powder samples were used for the VSM measurements (Micromag 3900, Princeton Measurement Corporation, Princeton, NJ, USA) and were used to determine the saturation magnetization of SPION^Cit^ and SPION^HAp^ at a field of 955 kA/m.

### 3.10. TEM Imaging and Selected Area Diffraction

TEM micrographs were taken using a Philips CM30 electron microscope (Philips, Netherlands), equipped with an LaB6 cathode, a twin objective lens (Cs = 2 mm), and an a1kx1k CCD camera. An acceleration voltage of 300 kV was used, providing a point resolution of 0.23 nm. SPION^HAp^ were diluted to a concentration of 5 μg Fe/mL and 10 μL dropped on carbon-coated copper grids (Plano, Wetzlar, Germany). As-prepared samples were dried under normal air conditions. The size distributions were calculated according to provided scale bars including 100 cores.

Diffraction patterns were obtained from the imaged nanoparticles. Images are provided with a scale bar that enables the direct determination of lattice plane spacing from the diffraction ring radius.

### 3.11. FTIR Measurements

FTIR measurements were recorded with an Alpha-P FTIR device (Bruker, Billerica, MA, USA) equipped with an ATR crystal. The wavelength range was 400–4000 cm^−1^ with a resolution of 4 cm^−1^, 128 sample scans, 64 background scans. OPUS software (Bruker, USA) was used for background subtraction and baseline correction.

### 3.12. Test for Endotoxin Contamination

Tests for the endotoxin measurements on particles were performed with an EndoZyme 2 Recombinant Factor C Assay kit (BioMérieux, Marcy-l’Étoile, France). In this kit, the endotoxin is detected by recombinant factor C, thus excluding possible interferences by β-glucans. Calibration curves were prepared in endotoxin-free water. Dispersions containing SPION^HAp^ at a concentration of 100 µg Fe/mL were diluted to a ratio of 1:10 and mixed with assay reagent (80% *v*/*v* assay buffer, 10% *v*/*v* substrate, 10% *v*/*v* enzyme) in a ratio of 1:1 and transferred into a black, endotoxin-free 96-well plate. The reaction was monitored for 90 min at 37 °C in 15 min intervals by recording the fluorescence at an excitation wavelength of 360 nm and an emission at 465 nm in a SpectraMax iD3 Plate reader (Molecular Devices, San José, CA, USA). The dilution was considered and the results were multiplied by a factor of 10. Spiking controls were carried out to assure that no interference occurred according to the assay manual.

### 3.13. Toxicological Tests

Three different cell lines were tested to confirm the toxicity effects of SPION^HAp^. All cells were passaged twice a week. Jurkat cells (ACC 282), which are non-adherent T cell leukemia cells, were used as a model system for toxicological tests. Cell culture of Jurkat cells was performed in RPMI-1640 supplemented with 10% FCS and 1% L-glutamine. EL4 (ATCC^®^ TIB-39™) cells were cultured in DMEM supplemented with 10% FCS and 1% L-glutamine and 1% penicillin-streptomycin. THP-1 monocytic cells (ATCC^®^ TIB-202™) were grown in RPMI-1640 supplemented with 10% FCS and 1% L-glutamine, with the addition of 1% penicillin-streptomycin. Incubation was carried out at 37 °C and humidified 5% CO2 atmosphere. A MUSE Cell Analyzer was used to determine the cell count.

To investigate the potentially toxic effects of SPION^HAp^ on cell viability, flow cytometry was used according to previously described methods [17,42,43,48]. Particles were dispersed in water and the pH was adjusted to 7.4. For this purpose, 3.6 × 10^4^ cells per well (48 well plate) of Jurkat cells and 2.0 × 10^4^ cells per well of EL4 (ATCC^®^ TIB-39™) and THP-1 (ATCC^®^ TIB-202™) cells were added to the wells in a volume of 360 μL media. To that volume, an amount of 40 μL SPIONs was added to reach a final concentration of 25 up to 400 μg nanoparticles/mL. Water served as a negative control and 2% DMSO as a positive control. In addition, EL4 and THP-1 were tested with 10 EU/mL of LPS from *Escherichia coli* O11:B4 and/or 0.1 µM dexamethasone as further controls. Each control and sample was measured in triplicate. Jurkat cells were incubated with the nanoparticles for 48 h under cell culture conditions. The staining solution contained 1 μL/mL Hoe of a 10 mg/mL solution, 2 μL/mL AxV, 66.7 ng/mL PI, and 0.4 μL/mL DiI of a 10 μM solution in Ringer’s solution. A total of 250 μL of the freshly prepared staining solution was added to 50 μL of the samples and controls. The stained samples were incubated under light protection at 4 °C for 20 min before analysis by flow cytometry (Gallios, Beckman Coulter, Brea, CA, USA). As excitation wavelength for AxV-FITC and PI 488 nm was set, the FITC fluorescence was recorded at 525/38 nm BP; PI was recorded at 620/30 BP; the DiI fluorescence was excited at 638 nm and recorded at 675/20 nm BP; Hoechst 33342 fluorescence was excited at 405 nm and recorded at 430/40 nm BP. Fluorescence bleed through was eliminated by electronic compensation. The acquired data were analyzed with the Kaluza software version 2.0 (Beckman Coulter).

### 3.14. Macrophage Culture and Cytokine Analysis

The bone marrow was isolated from 8- to 12-week old C57BL/6J mice (Janvier Labs, Le Genest-St-Isle, France) and differentiated into macrophages. Briefly, the femur and tibia were aseptically collected and the bone marrow was flushed out with sterile phosphate-buffered saline, pH 7.4 (PBS). After centrifugation, erythrocyte lysis was performed using ACK Buffer (150 mM NH_4_Cl, 10 mM KHCO_3_, 0.1 mM Na_2_EDTA, pH 7.4), the solution was neutralized with PBS and then filtered on a 70-µm cell strainer to remove clumps and cellular debris. The cell suspension was centrifuged again and resuspended in DMEM (Dulbecco’s Modified Eagle’s Medium) supplemented with 10% FCS and supplemented with 10% L929 cell-derived macrophage colony-stimulating factor supernatant. Cells were differentiated for 6 days prior to activation with SPION^HAp^ at an Fe concentration of 100 µg/mL, 10 EU/mL of LPS from *Escherichia coli* O11:B4 and/or 0.1 µM dexamethasone and incubated for 24 h. After incubation, cells were centrifuged and the supernatants recovered for measurement of secreted levels of TNF-α and IL-6 by enzyme-linked immunosorbent assay using the R&D System mouse DuoSet kit according to the manufacturer’s instructions (R&D Systems, Minneapolis, MN, USA).

### 3.15. Statistical Analysis

Statistical significance was examined by unpaired t-tests or a nonparametric Wilcoxon–Mann–Whitney test using GraphPad Prism 8.3.0. Asterisks were used to mark statistical significance: * *p* < 0.05, ** *p* < 0.005. *p* values higher than 0.05 were assumed not significant (n.s.).

## 4. Conclusions

In this study, we synthesized hydroxyapatite-coated iron oxide nanoparticles using citrate-coated SPIONs as precursors. The particles had a hydrodynamic diameter of 190 nm at neutral pH. The XRD, EDX, and FTIR results confirmed that the formation of a hydroxyapatite shell was successful. Importantly, the particles themselves were very well tolerated by EL4, THP-1, and Jurkat cells and induced only a minimal TNF-α and IL-6 release from mouse macrophages. At the same time, SPION^HAp^ potentiated the inflammatory and anti-inflammatory properties of co-stimulating biological molecules, such as LPS and dexamethasone, respectively. These findings, showing that cytokine release can be modulated by particles, can be potentially transferred to other particle systems, and utilized according to their potential applications. Specifically, higher levels of cytokines can be beneficial for mobilizing the immune system in certain therapeutic applications (e.g., cancer), whereas the suppression of cytokine release can be more useful to prevent escalating inflammatory conditions. Consequently, further research is required using SPION^HAp^ modified to serve different applications. Additionally, our findings suggest that cytokine release studies should be recommended for other particle systems, in order to avoid unwanted side effects upon nanoparticle administration in vivo.

## Figures and Tables

**Table 1:** Parameters of SPION^Cit^ and SPION^HAp^. Measurements were performed in triplicate.

	SPION^Cit^	SPION^HAp^
Z-average in nm	109 ± 1	190.7 ± 11.5
polydispersity index	0.189 ± 0.01	0.114 ± 0.02
ζ in mV	−46.9 ± 0.9	−28.1 ± 1.37

## Data Availability

The main data supporting the results of this study are available within the paper. The raw and analysed datasets generated during the study are available for research purposes from the corresponding authors on reasonable request.
